# Rapid screening for safety of donation from donors with central nervous system malignancies

**DOI:** 10.1097/MD.0000000000022808

**Published:** 2020-12-04

**Authors:** Mingxin Zhu, Yi Bian, Jipin Jiang, Ting Lei, Kai Shu

**Affiliations:** a Department of Neurosurgery; b Department of Emergency; c Institute of Organ Transplantation, Tongji Hospital, Tongji Medical College, Huazhong University of Science & Technology, Wuhan, Hubei, People's Republic of China.

**Keywords:** brain tumor, organ donation, selection, transmission

## Abstract

With the increasing demand on organ transplants, it has become a common practice to include patients with primary central nervous system (CNS) malignancies as donors given the suggested low probability metastatic spread outside of the CNS. However, an extra-CNS spread of the disease cannot be excluded raising potential risks of cancer transmission from those donors. In order to balance between the risk of donor-derived disease transmission and the curative benefit for the recipient, a careful donor and organ selection is important. We performed a literature research and summarized all reported studies of organ transplants from donors suffered from primary CNS malignancies and determined the risk of tumor transmission to recipients. There were 22 cases of transplant-transmitted CNS tumors onto recipients since 1976. The association risks of cancer transmission were attributed to donor tumor histology, disruption of the blood-brain barrier, cerebrospinal fluid extra-CNS, and false diagnosis of primary intracranial tumor as well as the molecular properties of the primary tumor such as the existence of EGFR-amplification. The association risks and features of CNS tumors transmission recipients indicated that we need to reassess our thresholds for the potential fatal consequences of these donors.

## 1 Introduction

In global solid organ transplantation there is an increasing discrepancy of available supply and awaiting receipting patients with over 50,000 patients waiting in the European Union, 120.000 patients waiting in the United States and more than 300,000 waiting patients in China.^[1–3]^ The mismatch between organ resources and demand remains despite of the increasing number of organs derived from deceased donors. In order to expand the pool of organs available for transplantation, it has become a common practice to accept organs from donors suffering from low grade tumors and tumors that have been diagnosed as “low metastatic risk”.^[4,5]^ For decades, patients with primary central nervous system (CNS) tumors are regarded as relatively safe resources for organ donations because of the significantly low incidence rate of extra-CNS spread.^[6,7]^ Recent published guidelines propose that even organs form patients dying of primary CNS tumors including those with high WHO malignancy grade tumors, should be considered for organ donation according to the data from the UK Transplant Registry (UKTR), United Network for Organ Sharing (UNOS) and the Australia and New Zealand Organ Donation Registry (ANZODR).^[8–10]^

The overall risk of cancer transmission from organ donors with CNS malignancies seems limiting low. However, since 1976, there have been at least some dozens of cases of transmission of primary CNS tumors to transplant recipients.^[8,11–13]^ In early beginning in neuro-oncology, physicians recognized that the general estimates of extra-CNS spread have been quoted less than 1%.^[14,15]^ These studies depend on the fact that the physiological normal brain is anatomically and immunologically privileged and separated as compared to the rest of the body through the existence of the filering system of blood brain barrier. With the recent technical advances in liquid biopsy, the detection of predictive biomarkers in the blood such as circulating cancer cells, circulating tumor DNA or extracellular vesicles such as episomes or serum metabolites have emerged.^[16–18]^ Although their importance in establishing extra-cerebral micro-metastatic sites is not mechanistically proven, they propose a potential risk of transmitting the disease to distant organs. As such, Muller and colleagues analyzed 141 patients with glioblastoma and identified circulating tumor cells in 29 cases.^[19]^ Sullivan et al. reported that in 39% of all glioblastoma cases they could detect circulating tumor cells. It is believed that, despite the high infiltrative and invasive properties of the primary tumor, the tumors are so highly malignant that the patients die before establishing metastatic sites outside the brain.^[7,20]^ It is unknown whether, besides not having established outside-CNS metastasis, those patients may already be in a stage of systemic tumor spread and thereby oppose a significant risk for disease transmitters in case of organ donation.

This review will discuss the transmission features, association risks, possible mechanism and evaluation protocols of transplantation on the use of organs from donors with primary CNS malignancies which should help the physicians and regulatory authorities to better assess the potential risks with such donor cohorts.

## 2 Methods

The primary objective of this study was to analyze clinical characteristics, association risks and outcome of cancer transmission on the use of organs from donors with primary brain tumors. First, a system literature search strategy was conducted using PubMed and Web of Science using the following search entries: “central nervous system tumor” AND “organs” AND “transmission” AND “recipients”. Only cases of primary-to-primary tumor transmission was included, cases of development of secondary brain tumors in recipients were excluded in the analyzes. Twenty-two cases of cancer transmission from patients with malignant primary brain tumor published were compiled since 1976. In total, a total of nineteen papers including case reports and reviews were analyzed and included in this study.

From the published reports, the following data were collected:

(1) year of publication,
(2) age of patient,
(3) pathology type of transmission tumor,
(4) time to metastasis following transplantation,
(5) overall survival (OS) following transplantation,
(6) treatment received as categorized by craniotomy and/or radiation and/or chemotherapy, and/ or cerebrospinal fluid shunting.

**Figure 1:**
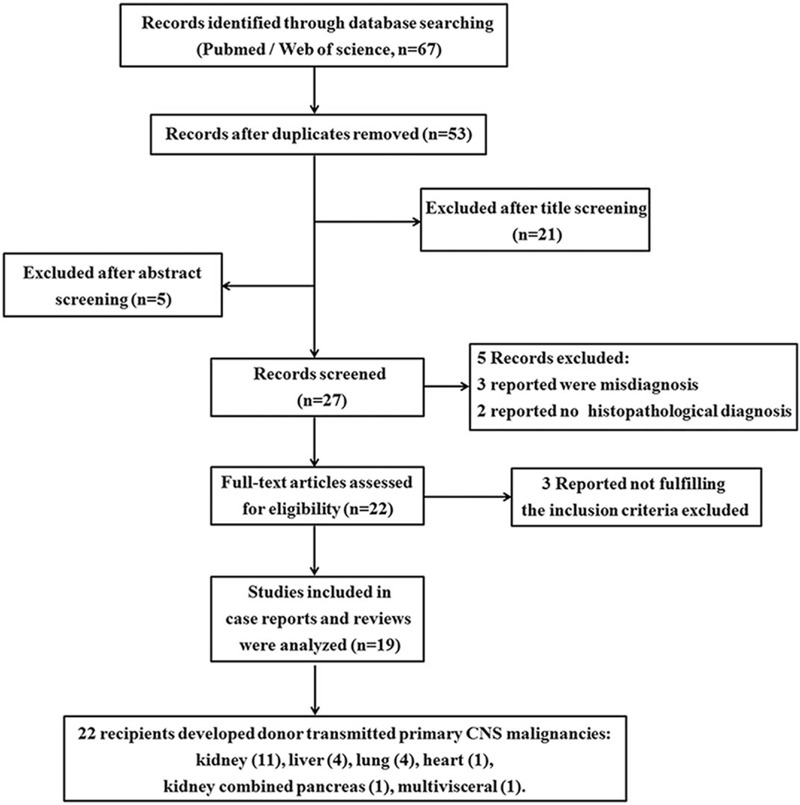
Procedure of publication retrieval and exclusion of cases. A PRISMA flow chart for systematic review of cancer transmission from organs of donors with central nervous system malignancies.

Donor's prior risk factors and recipient survival rates were also evaluated. Moreover, the time to metastasis and OS were included in our analysis. The procedure of publication data retrieval and inclusion in evaluation as well as exclusion of cases is displayed in flow chart depictured (Fig. 1).

## 3 Results

### 3.1 Quantification of recorded transmissions of CNS malignancies

Three donor registries have reported their experience: UKTR data showed no case of transmission of donor-transmitted malignancies out of 179 donors with a history of primary intracranial malignancy donating to 448 recipients. ANZODR data reported 151 recipients of 46 donors with primary CNS tumors and no case of transmission of donor malignancy was identified. The UNOS and the Organ procurement and Transplantation Network reported 3 recipients had transmitted malignancies from 642 donors with primary CNS tumors. According the retrospective studies performed by the databases, there were at least hundreds of donors with primary CNS malignancies that provided their organs for organ transplantation. Moreover, there was 22 cases recorded tumor transmission of donor-transmitted malignancies from 1976 to 2014.^[12,21–35]^

By the data of these reports, the grafted organs with primary CNS malignancies transmission included 11 cases of kidney transplantation, 4 cases of liver transplantation, 4 cases of lung transplantation, 1 case of heart transplantation, 1 case of kidney combined with pancreas transplantation and 1 multi-visceral transplantation.

### 3.2 Clinical course of patients with recorded tumor transmission

The time to metastasis and medium OS as a result of transmission of primary CNS malignancies were analyzed. 22 recipients were transplanted from 16 organ donors with primary CNS malignancies. The average time to metastasis for kidney transplants (n = 7 cases) was 13.2 months post transplantation with an medium OS of 29.6 months. For liver transplant patients (n = 4 cases), three patients died at an average of 7 months post-surgery with a mean time to metastasis of 5.7 months. All lung transplant recipients (n = 4 cases) succumbed due to glioblastoma metastasis. The average OS was 12.5 months (n = 3 recorded cases). One patient was diagnosed with brain cancer metastasis 4 months after receiving multivisceral transplantation and died 6 month post-surgery. Table 1 list OS rates and time to metastasis for all recipients.

**Table 1:** Prognosis of recipient transmitted with donor CNS malignancies.

Reference	Recipient's age and gender	Transplanted organ	Time to metastasis (months)	Overall survival time (months)	Pathology type of transmission tumor	Year
Barnes et al	61y, M	Kidney	4.5	5	Malignant Glioma	1976
Barnes et al	38y, M	Kidney	4.5	>17	Malignant Glioma	1976
Lefrancois et al	N/A	Kidney	N/A	N/A	Medulloblastoma	1987
Lefrancois et al	N/A	Kidney and Pancreas	N/A	N/A	Medulloblastoma	1987
Lefrancois et al	N/A	Heart	N/A	N/A	Medulloblastoma	1987
Morse et al	44y, F	Liver	8	10	Malignant Glioma	1990
Konigsrainer et al	N/A	Kidney	N/A	N/A	Lymphoma	1993
Konigsrainer et al.	N/A	Kidney	N/A	N/A	Lymphoma	1993
Ruiz et al	48y, F	Kidney	17	>33	Glioblastoma	1993
Ruiz et al	23y, M	Kidney	18	>33	Glioblastoma	1993
Val-Bernal et al	48y, F	Kidney	17	> 36	Glioblastoma	1993
Colquhoun et al	32y, M	Kidney	10	N/A	Glioblastoma	1994
Colquhoun et al.	23y, F	Kidney	10	N/A	Glioblastoma	1994
Jonas et al	28y, F	Liver	4	6	Glioblastoma	1996
Bosmans et al	49y, M	Kidney	12	>24	Meningioma	1997
Frank et al	29y, N/A	Liver	N/A	5	Glioblastoma	1998
Armanios et al	28y, M	Lung	4	4.5	Glioblastoma	2004
Chen et al	57y, M	Lung	14	17	Glioblastoma	2008
Fatt et al	58y, M	Lung	12	N/A	Glioblastoma	2008
Kashyap et al	54y, M	Liver	5	N/A	Malignant Astrocytoma	2009
Zhao et al	17m, M	Multivisceral	4	6	Pineoblastoma	2012
Nauen et al	57y, M	Lung	15	16	Glioblastoma	2014

CNS = central nervous system.

### 3.3 The probability and association risks of CNS malignancies patients

The histologic distribution of tumors identified in the 16 donors predominantly identified glioblastomas or gliomas (n = 11 cases), 1 astrocytoma, 1 medulloblastoma, 1 non-hodgkin's lymphoma, 1meningioma, and 1 pineoblastoma. These 16 donors provided organs to 35 recipients, 22 of whom developed donor transmission tumor or in other words, in 62% of the transplantation of organs a transmission was recorded. The risk of cancer transmission is alarming and caution must be taken when including such cohorts in organ donation campaigns. The information of these donors were provided, and all most of them had at least 1 risk factor, including the diagnosis of high WHO grade brain tumors (n = 14), craniotomy (n = 7), radiation therapy (n = 4) and tumor apoplexy or/ and hemorrhage (n = 3), ventricular shunt (n = 1). The list of risk factors is provided in Table 2.

**Table 2:** Association risks from organ donors.

				Risk factors			
Year	Reference	Donor's age and gender	Pathology type of CNS tumors	Ventricular shunt	Craniotomy	Radiation therapy	Tumor apoplexy / hemorrhage
1976	Barnes et al	45yr, M	Malignant Glioma	No	No	No	No
1987	Lefrancois et al	N/A	Medulloblastoma	Yes	Yes	Yes	No
1990	Morse et al	14yr, M	Malignant Glioma	No	No	No	No
1993	Konigsrainer et al	70yr, N/A	Non-Hodgkin's Lymphoma	N/A	N/A	N/A	N/A
1993	Ruiz et al	42yr, M	Glioblastoma	No	Yes	Yes	No
1993	Val-Bernal et al	42yr, M	Glioblastoma	No	Yes	Yes	No
1994	Colquhoun et al.	32yr, M	Glioblastoma	No	Yes	No	No
1996	Jonas et al	48yr, F	Glioblastoma	N/A	Yes	N/A	N/A
1997	Bosmans et al	N/A	Meningioma	N/A	N/A	N/A	N/A
1998	Frank et al	47yr, F	Glioblastoma	No	Yes	No	No
2004	Armanios et al	29yr, M	Glioblastoma	No	Yes	Yes	Yes
2008	Chen et al	47yr, M	Glioblastoma	No	No	No	Yes
2008	Fatt et al	58yr, M	Glioblastoma	No	No	No	N/A
2009	Kashyap et al	27yr, F	Astrocytoma	No	N/A	N/A	Yes
2012	Zhao et al	14mo, M	Pineoblastoma	No	No	No	No
2014	Nauen et al	N/A	Glioblastoma	N/A	N/A	N/A	N/A

## 4 Discussion

Primary CNS malignancies incidence is approximately 5.26 to 7 per 100,000 individuals per year in worldwide.^[36–38]^ An average of just more than 7500 patients aged younger than 65 years old die of primary CNS malignancies annually^[13,39]^ and currently there is no risk management in terms of including such diseased patients in the organ donor routine.^[5,6]^ The UNOS/ Organ Procurement Transplantation Network data indicated that annually approximately 50 to 60 patients were procured.^[13]^ Of note, in the United Kingdom, more than four hundreds recipients had received organs from the donors who were died of primary CNS malignancies.^[8]^ These results indicated that the recipients who had been transplanted organs from primary CNS malignancies provided a longer survival when compared with the patients who underwent long-term waiting until transplantation or even none transplantation.^[5,40]^

Data of UKTR and ANZODR showed that there was no case of donor-derived transmission of metastatic malignancy.^[8,9]^ In contrast, a retrospective review of the Israel Penn International Tumor Registry reported that nearly 23% recipients of organs originating from patients with brain cancer developed donor- transmitted tumors.^[41]^ Furthermore, other institutional data reported the risk of transmission from donors with CNS malignancies to recipient was rare.^[13,26,27]^ Reliable data on the actual percentage of cancer transmission significantly varies in between registries. Our search identified 22 cases of transplant transmitted disease since 1976. A consensus guideline, defining minimum features to be recorded of each organ of a donor with brain cancer and the donor itself, are needed.

Our understanding how genetic apparitions and mutations increases brain cancer malignancy increases rapidly. Molecular subclassification of brain cancer revolutionizes neuropathology, however, the development of adequate treatment options is lagging^[42]^ behind. In the wake of extended molecular profiling of brain cancers during routine clinical diagnostics, our understanding of any genetic association of the tumor to predict for high risk transmission capacities will improve. Current research identified scientific paradigms of tumor stemness and mesenchymal transformation in glioblastoma as key drivers for invasion. It would be interesting to validate any association of elevated stemness markers and mesenchymal differentiation status in the tumor to possible altered frequency of occurrence of extra-CNS transmission.^[43,44]^

The brain's lack of lymphatic drainage system and the blood brain barrier represent barriers for extra-CNS spread of tumor cells. However, most cases with cancer transmission were found when the patient had a disrupted of the blood brain barrier due to a consequence of current standard of care treatment or as a results of the tumor growth itself.^[13,19,45]^ In additional, a part of cases develop hydrocephalus which means the presence of ventriculoatrial shunts as well as ventriculoperitoneal shunts could be regarded as a risk factor for cancer transmission.^[5,8]^ Resent reports found that extracranial GBM metastases occur often in the pleura and lungs, liver, bone marrow and regional lymph nodes.^[7,46,47]^ Misdiagnosis of donor brain death due to primary intracranial tumor also has fatal consequences immediately leading to increasing risk of cancer transmission to recipients. Buell et al. previously reported that 23% (14/62) incidences of tumor transmission from organ donors with CNS malignancies.^[41]^ However, in the following studies they found that there were 42 recipients transplanted with organs from donors with misdiagnosed primary CNS tumors. After transplantation these donors were identified with variable tumors including melanoma (23%), renal cell carcinoma (19%), and choriocarcinoma (12%).^[48]^ Misdiagnosis CNS tumors included in the pool of possible donors are associated with high mortality and high transmission rates: Donors without definitive histopathological diagnosis should be avoided for transplantation. Recent studies reported that glioblastoma, the most common and aggressive primary malignant brain tumor, may metastasize outside of the CNS system. Accessing via dural vessels to extrameningeal tissue is considered the most likely path in the development of metastases. Evidence supporting this mechanism based on the pattern of seeding in lymph modes and the lungs, which are the most frequent affected organs. All of these suggested that either lymphatic or hematologic routes is of importance. However, the infiltrative feature of this aggressive brain cancer seldomly leads to the establishment of extracranial cancer sites, with only about 0.4% to 2.0% of patients developing secondary tumors outside of the brain.^[17,46]^ Seoane et al. discussed several reasons: (i) the cancer cells are not able to thrive on extracranial niches; (ii) the cancer cells might require some brain-specific growth factors; and (iii) the short survival periods of patients with GBM do not allow sufficient time to develop metastasis.^[17]^ Further reports elucidated that in 20% up to 40% of glioblastoma patients with tumors characterized by EGFR-amplification are positive for the existence of circulating tumor cells in the peripheral blood.^[17,19,49]^ The authors observed that the glioblastoma cells with EGFR copy gain were more prone to disseminate to the bloodstream than other tumor cell clones. These studies indicated that EGFR-amplification in WHO high grade primary brain tumors predict for a higher risk of outside-the-brain metastatic capacity supposingly contributing to extracranial transmission.^[50,51]^

**Figure 2:**
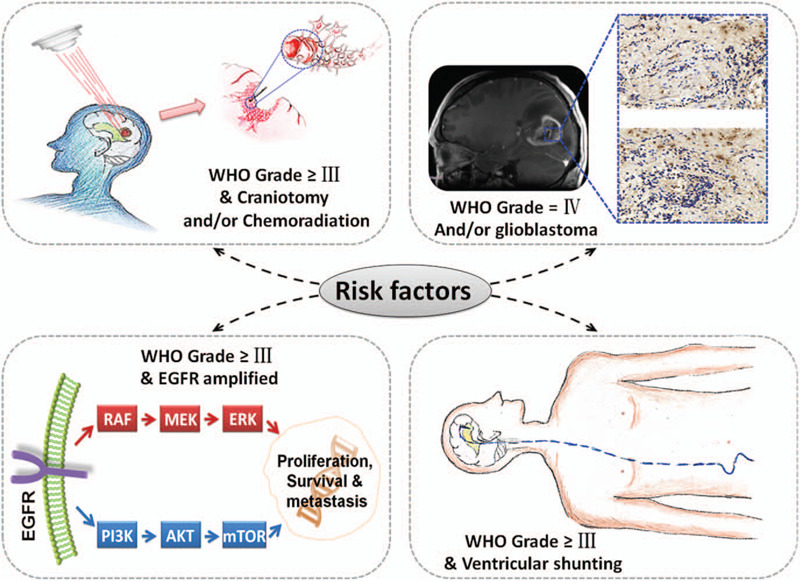
The probability of CNS malignancies transmission. The association risks of transmission from donors to recipients has been attributed to donor tumor histology (WHO grade IV), disruption of the blood-brain barrier (craniotomy and chemoradiation therapy), cerebrospinal fluid extra-CNS (ventriculoatrial and ventriculoperitoneal shunts), as well as natural characteristics of tumor (high level of EGFR-amplified). The more related factors, the more risk of cancer transmission.

**Figure 3:**
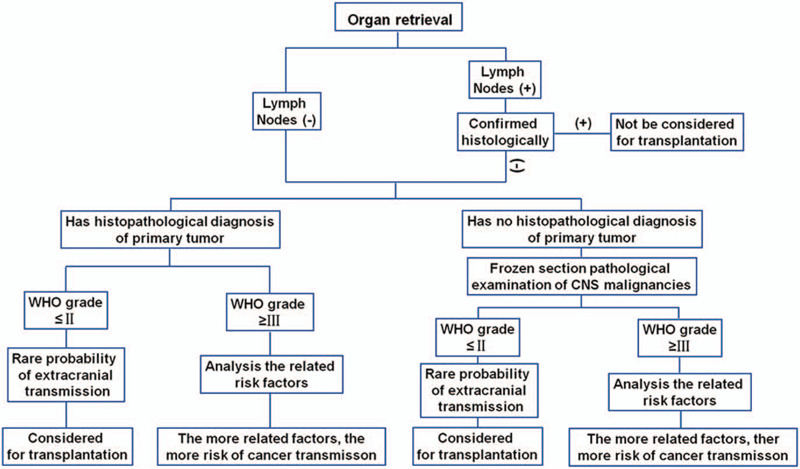
Flow chart for donor selection.

We hypothesize that the listed “risk factors for cancer transmission” (Fig. 2) could be included and further develop a consensus how to predict the risk of organ transplantation-mediated tumor transmission (Fig. 3).

## 5 Conclusion

These reports are consistent with those in our study, which demonstrated that the possibility of malignant tumors transmission from CNS tumors donors was very rare. Kauffman et al. reported that, in the United States, the death rate owing to donor-related tumors was extremely low at 0.007%.^[40]^ Our studies emphasize that, although rare, brain cancer transmission through organ donation is possible even if it is rare. With our advances in understanding of molecular subtypes in neuro-oncology, a concise monitoring should aim to define novel “transmission risk markers” which, in conjunction with described “risk markers,” will help to better select safe donor candidates. With increasing demand on organ on increasing global transplantation campaigns, our study should highlight the importance of case-by-case decision making to avoid spreading of otherwise non-infectious lethal diseases.

## Author contributions

**Data analysis**: Mingxin Zhu, Yi Bian, Ting Lei and Kai Shu.

**Data curation:** Yi Bian, Ting Lei.

**Formal analysis:** Jipin Jiang.

**Investigation**: Mingxin Zhu, Jipin Jiang, and Ting Lei

**Manuscript preparation**: Mingxin Zhu, Yi Bian and Kai Shu

**Writing – review & editing:** Yi Bian, Ting Lei.
